# Supplementary material for: A UMLS-based spell checker for natural language processing in vaccine safety
Source: BMC Med Inform Decis Mak. 2007 Feb 12;7:3. doi: 10.1186/1472-6947-7-3 (PMC1805499; doi:10.1186/1472-6947-7-3)
Supplement: Additional file 13 — RAP application directory. Sets up directory for RAP (RDF application for PHP) source code [file 1472-6947-7-3-S13.gz › rap/doc/phpdoc/li_infModel.html]

xml version="1.0" encoding="iso-8859-1"?


infModel

Description
:   Class trees  
    Index of elements  
    Todo List

Classes
:   InfModel
:   InfModelB
:   InfModelF
:   InfRule
:   InfStatement

Files
:   InfModel.php
:   InfModelB.php
:   InfModelF.php
:   InfRule.php
:   InfStatement.php

phpDocumentor v 1.3.0RC3
